# Medical Potential of Insect Symbionts

**DOI:** 10.3390/insects16050457

**Published:** 2025-04-26

**Authors:** Fanglei Fan, Zhengyan Wang, Qiong Luo, Zhiyuan Liu, Yu Xiao, Yonglin Ren

**Affiliations:** 1 School of Food and Strategic Reserves, Henan University of Technology, Zhengzhou 450001, China; 2023920016@stu.haut.edu.cn (F.F.); zywangedu@163.com (Q.L.); 2022920138@stu.haut.edu.cn (Z.L.); 2 College of Environmental and Life Sciences, Murdoch University, Perth, WA 6150, Australia; yu.xiao@murdoch.edu.au

**Keywords:** antibiotic, antiparasitic activity, antitumor activity, insect symbiont, medical application, secondary metabolite

## Abstract

Insect symbionts and their metabolites are complex and diverse and are gradually becoming an important source of new medical materials. Some culturable symbionts from insects produce a variety of active compounds with medical potential. Among them, fatty acids, antibacterial peptides, polyene macrolides, alkaloids, and roseoflavin can inhibit the growth of human pathogenic bacteria and fungi; lipases, yeast killer toxins, reactive oxygen species, pyridines, polyethers, macrotetrolide nactins, and macrolides can kill human parasites; and peptides and polyketides can inhibit human tumors. However, due to difficulty in the culture of symbionts in vitro, difficulty in targeting bacteria to specific sites in the human body, the limited capability of symbionts to produce active metabolites in vitro, inconsistent clinical research results, adverse reactions on humans, and the development of antibiotic resistance, the application of insect symbionts and their metabolites in the medical field remains in its infancy. This paper summarizes the medical potential of insect symbionts and their metabolites and analyzes the status quo and existing problems with their medical application. Possible solutions to these problems are also proposed, with the aim of hastening the utilization of insect symbionts and their metabolites in the medical field.

## Simple Summary

Insects have evolved close associations with microbial symbionts. Insect symbionts produce a variety of bioactive compounds with medical potential, such as fatty acids, peptides, polyethers, and polyketides, which show activity in inhibiting human pathogenic bacteria and fungi, killing parasites, and suppressing tumors. However, the application of these symbionts and their metabolites in the medical field remains in its infancy, which can be attributed to technological limitations and multiple challenges in research and application. These challenges can be addressed by utilizing insect cell cultures, screening new symbiont strains and metabolites, combining multiple symbiont strains, combining symbionts with synergists, and conducting adverse reaction surveillance and prediction. The paper aims to enhance the discovery, production, and safe application of insect symbionts and metabolites in the medical field, regarding them as a promising bridge to novel and effective therapeutic agents.

## 1. Introduction

The Insecta, accounting for over 80% of all arthropods, colonizes almost all the global ecological niches [1]. Insects have evolved close associations with microbial symbionts [2]. Insects provide the living environment for their symbionts, while the symbionts affect host physiology and behavior, including development, reproduction, nutrition, pathogen defense, toxin metabolism, and chemical communication [3]. For example, insect symbionts can produce antibacterial substances, regulate host immune-related genes, and compete with exotic microorganisms for ecological niches, thereby protecting the host from pathogen invasion [4]. These immune-related insect symbionts are new sources for screening microbial strains with great medical value.

The medical potential of insect symbionts has gradually been validated in many studies. Culturable symbionts isolated from insects can be ingested into the human body and exert positive influences on human physiology. *Apilactobacillus kunkeei* from the gut of the honeybee *Apis mellifera* converts fructose into mannitol and regenerates NAD^+^, enabling metabolism to continue. This ameliorates fructose-mediated irritable bowel syndrome [5,6]. Lactic acid bacteria isolated from the silkworm *Bombyx mori* interfere with the recycling of bile salts, metabolic products of cholesterol, and facilitate their elimination. They also bind and fix cholesterol and inhibit the production of cholesterol in the blood, thus reducing the incidence rate of cardiovascular and cerebrovascular diseases [7].

In addition, metabolites from insect symbionts exhibit activity against bacterial infections, parasites, and tumor cells, which threaten human health and life. Nodupetide discovered from *Nodulisporium* sp. IFB-A163, a fungus residing in the gut of the bean bug *Riptortus pedestris*, inhibits the growth of the drug-resistant human pathogenic bacterium *Pseudomonas aeruginosa* [8]. Paecilodepsipeptide A, produced by *Paecilomyces cinnamomeus* BCC 9616, associated with scale insects, demonstrates potent inhibition of the malaria parasite *Plasmodium falciparum* K1 [9]. A new spectinabilin derivative related to the ant symbiont *Streptomyces* sp. 1H-GS5 is found to have antitumor activity [10].

The most promising insect symbionts with medical potential are gut symbionts [11]. However, their exploitation is currently hindered by some issues, such as difficulty in the culture of insect symbionts in vitro and negative effects of probiotics on humans [12,13,14]. Therefore, the application of insect symbionts in the medical field remains in its nascent phase [15]. Given the current state of research, this paper aims to provide a comprehensive overview of the medical potential of insect symbionts and their metabolites. The ultimate goal is to accelerate the utilization of insect symbionts and their metabolites in the medical field.

## 2. Medical Potential of Insect Symbionts

### 2.1. Inhibition of Human Pathogenic Bacteria and Fungi

**Figure 1:**
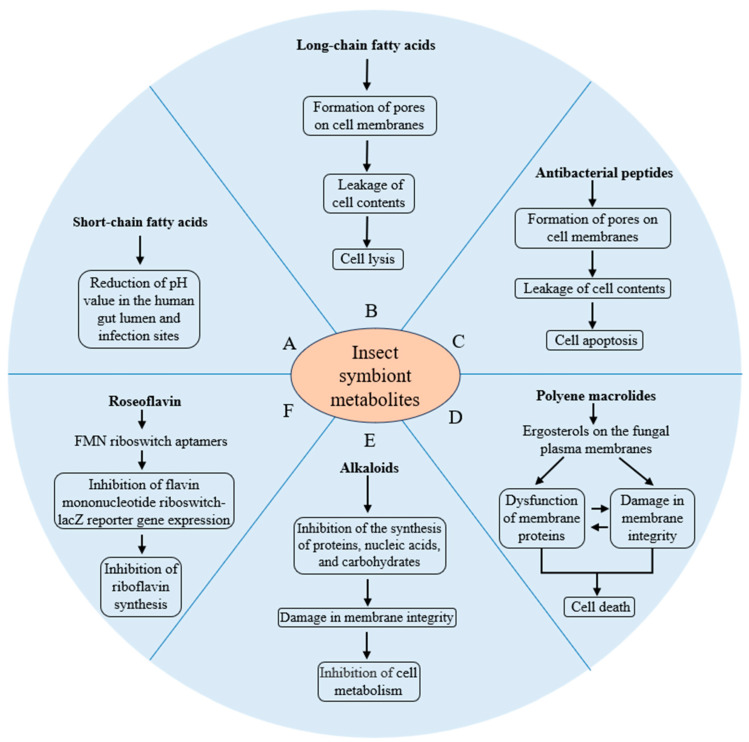
Effects of insect symbiont metabolites on pathogenic bacteria and fungi. (A) Effects of short-chain fatty acids on pathogenic bacteria; (B) Effects of long-chain fatty acids on pathogenic bacteria; (C) Effects of antibacterial peptides on pathogenic bacteria; (D) Effects of polyene macrolides on pathogenic fungi; (E) Effects of alkaloids on pathogenic bacteria; (F) Effects of roseoflavin on pathogenic bacteria. Arrows depict activation in interactions.

Insect symbionts produce antibacterial or antifungal substances in the form of fatty acids, antibacterial peptides, polyene macrolides, alkaloids, and roseoflavin (Table S1) (Figure 1). The antibacterial or antifungal activity of these substances is often observed in in vitro experiments.

#### 2.1.1. Fatty Acids

Short-chain fatty acids, such as lactic, oxalic, glutaric, and acetic acids, produced by lactic acid bacterial strains isolated from the gut and stomach of *A. mellifera* can inhibit the growth of pathogenic bacteria including *Escherichia coli*, *Salmonella typhimurium*, and *Staphylococcus aureus* [16,17]. The pH of the lactic acid bacterial suspension is around 4–5 [16], and the pH gradually increases in the human small intestine from pH 6 to pH 7.4 in the terminal ileum [18]. A decrease in pH caused by short-chain fatty acids produced by lactic acid bacteria can be an inhibiting factor for the growth of bacteria in the human intestine [16].

The carbon chain of long-chain fatty acids has a carboxyl group at one end and a hydrocarbon chain at the other. The carboxyl group is hydrophilic and ionized when solubilized in water, whereas the hydrocarbon chain is hydrophobic, making the entire molecule amphipathic [19]. Some of the detrimental effects of long-chain fatty acids on bacterial cells can be attributed to the detergent properties of long-chain fatty acids on account of their amphipathic structure. This allows them to specifically interact with the cell membrane of bacteria to create transient or permanent pores of variable sizes, thereby leading to the leakage of cell contents and ultimate cell lysis [20,21].

**Figure 2:**
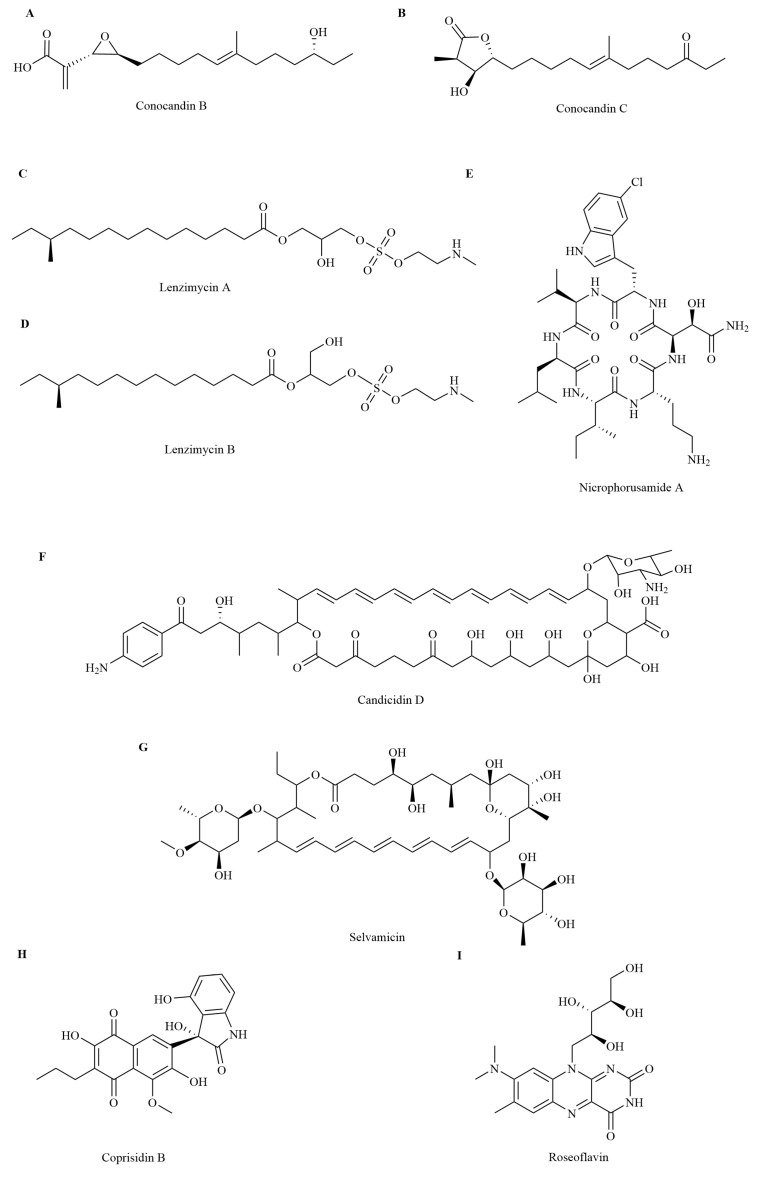
A selection of antibacterial compounds isolated from insect symbionts. (**A**) Conocandin B; (**B**) Conocandin C; (**C**) Lenzimycin A; (**D**) Lenzimycin B; (**E**) Nicrophorusamide A; (**F**) Candicidin D; (**G**) Selvamicin; (**H**) Coprisidin B; (**I**) Roseoflavin.

Conocandins and lenzimycins are novel antibacterial long-chain fatty acid derivates isolated from the fungal and bacterial microbiomes that are associated with insects. *Pseudonocardia* sp. isolated from the nests of the fungus-growing ant *Trachymyrmex* sp. produces conocandins B and C (Figure 2A,B), which inhibit the growth of *Escovopsis* sp. [22]. Lenzimycins A and B (Figure 2C,D) produced by symbionts (*Brevibacillus* sp. PTH23) of the dung beetle *Onthophagus lenzii* inhibit the growth of *Bacillus* sp. CCARM 9248, *Enterococcus faecium*, and certain strains of *Enterococcus faecalis* [23].

The advantage of long-chain fatty acids as antibacterial agents is that they are non-toxic and safe to humans [24]. Furthermore, long-chain fatty acids produced by insect symbionts activate host immune pathways, which affect host lipid metabolism and enhance host fitness. Therefore, fatty acids from insect symbionts have potential benefits for human health [25].

#### 2.1.2. Antibacterial Peptides

Antibacterial peptides have an amphiphilic helical structure, which allows them to insert into the membrane of the target cell. The insertion of antibacterial peptides causes pore formation in the cell membrane of the target pathogenic bacteria, thereby leading to the depolarization of the cytoplasmic membrane, leakage of cell contents, and cell apoptosis [26,27]. For example, the structure of the hydrophilic hydroxy group in *D*-*threo*-*β*-hydroxyasparagine of nicrophorusamide A (Figure 2E), produced by *Microbacterium* sp. isolated from the gut of the carrion beetle *Nicrophorus concolor*, makes a rigid conformation with the corresponding hydrogen bond and enhances the interaction between nicrophorusamide A and the target cell membrane. Nicrophorusamide A exhibits antibacterial activity against *S. aureus* and *E. faecalis* [28].

Antibacterial peptides have clear advantages over conventional antibiotics, including slower emergence of resistance, broad-spectrum antibiofilm activity, and the ability to favorably modulate the host immune response [29]. Methods used to screen natural antibacterial peptides are not well developed. For example, the screening method of whole-bacterial adsorption binding, which depends on the interactions between antibacterial peptides and bacterial cell membranes, has limitations in sensitivity [30].

#### 2.1.3. Polyene Macrolides

The polyene macrolides with multiple conjugated double bonds and macrolide skeletons are important antifungals used for treating topical and systemic infections, particularly those caused by *Candida*, *Cryptococcus*, and *Aspergillus* [31]. Polyene macrolides interact with ergosterol, the primary sterol of fungal plasma membranes, compromising membrane integrity, and inhibit the function of membrane proteins, leading to cell death [32]. This class of antimicrobial molecules has been recurrently identified in microbial symbionts of various fungus-growing ants. *Streptomyces* spp., isolated from the body surface and fungus gardens of *Acromyrmex octospinosus*, produce candicidin D (Figure 2F), which strongly inhibits the growth of the fungus *Escovopsis weberi* [33,34]. Selvamicin (Figure 2G), produced by *Pseudonocardia* sp. isolated from *Apterostigma* ant nests, inhibits the growth of *Candida albicans* [32]. The polyene macrolides selectively inhibit the growth of fungi rather than bacteria, and there is a minimal chance of the development of microbial resistance to them [35].

#### 2.1.4. Alkaloids

Alkaloids are nitrogen-containing heterocyclic compounds that inhibit the growth of bacteria and lead to the inactivity of biofilm-related bacteria cells through various mechanisms [36]. They exhibit strong inhibitory effects on the synthesis of proteins, nucleic acids, and carbohydrates in bacteria, thereby destroying cell membrane permeability and cell membrane and cell wall structure and inhibiting bacterial metabolism and efflux pumps [37]. The naphthoquinone alkaloid, coprisidin B (Figure 2H), is purified from *Streptomyces* sp. SNU607, which is isolated from the gut of the dung beetle *Copris tripartitus.* Coprisidin B inhibits sortase A, a virulence factor in *S. aureus* that participates in the establishment and persistence of infections [38]. Alkaloids have the characteristics of a broad antibacterial spectrum, fewer adverse reactions, and a low tendency to antibiotic resistance [36].

#### 2.1.5. Roseoflavin

Roseoflavin (Figure 2I) is a structural analog of riboflavin (vitamin B_2_) and flavin mononucleotide [39]. Roseoflavin produced by *Streptomyces davaonensis* YH01 that is isolated from the body surface of the queen of the fungus-growing termite *Odontotermes formosanus* shows strong inhibitory activity against *Bacillus subtilis* and *S. aureus*. The antibacterial mechanism of roseoflavin is mainly due to the potential riboswitch inhibition of bacteria. Roseoflavin can directly bind to flavin mononucleotide riboswitch aptamers and downregulate the expression of the flavin mononucleotide riboswitch-lacZ reporter gene in *B. subtilis*, thereby inhibiting the synthesis of riboflavin, which is essential for the growth of pathogenic bacteria [40]. Roseoflavin is a natural antibacterial substance with stable characteristics. However, many roseoflavin-resistant strains of *B. subtilis* and some other Gram-positive bacteria have been identified and characterized [39].

### 2.2. Inhibition of Parasites

#### 2.2.1. Inhibition of Malaria Parasites

Malaria is a disease resulting from the continuous proliferation of the parasite *Plasmodium* in the human erythrocyte. Infections may induce serious anemia, acute kidney injury, and cerebral malaria [41]. Malaria parasites infest humans via the bite of infected female *Anopheles* mosquitoes [42]. The life cycle of the parasite in the mosquito midgut begins with the activation of the intraerythrocytic gametocytes instantly after ingestion and concludes with the traverse of the midgut epithelium by the invasive ookinetes within 24 h after infestation. Subsequently, the ookinetes colonize the basal layer of the midgut epithelium and develop into sessile oocysts, in which sporogonic replication occurs and infective sporozoites are formed [43].

In the six Southeast Asian countries that constitute the Greater Mekong Subregion, resistance to derivatives of artemisinin, which are principal components of first-line malaria treatments, has been found in *P. falciparum*. Thus, it is essential to find new compounds to kill malaria parasites [44]. Symbionts that inhabit the gut of the mosquito generate lipases, yeast killer toxins (a kind of protein), and reactive oxygen species to kill malaria parasites directly or indirectly. *Serratia ureilytica* Su_YN1 separated from *Anopheles sinensis* suppresses the growth of *Plasmodium* by secreting a *Plasmodium*-killing lipase [42].

**Figure 3:**
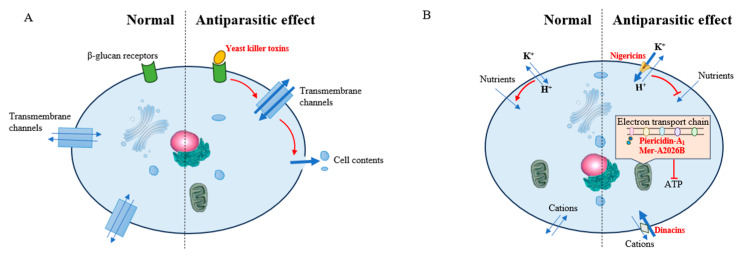
Inhibition of the growth of the malaria parasite (**A**) and *Leishmania donovani* (**B**) by insect symbiont metabolites. Red arrows depict activation, while red bars represent suppression in molecular interactions. Thicker lines represent high flow rates. Dashed lines divide the cells into the normal half and the half in which the metabolites of insect symbionts exert antiparasitic effects.

A yeast killer toxin, generated by *Wickerhamomyces anomalus* separated from *Anopheles stephensi*, is an effector molecule which targets the sporogonic stages of the malaria parasite *Plasmodium berghei* in vitro. The yeast killer toxin binds to β-glucans, which are specific receptors situated on the surface of the cell wall of *P. berghei*. The binding of the yeast killer toxin to the membrane receptor induces the formation of transmembrane channels, resulting in the leakage of intracellular contents and intensely suppressing parasite development from gametocytes to ookinetes [45,46] (Figure 3).

*Enterobacter* sp. Esp_Z separated from wild-type mosquito populations in Zambia increases the resistance of the mosquito to infections by the parasite *P. falciparum.* It achieves this by disrupting parasite development before invasion of the midgut epithelium. This anti-*Plasmodium* effect is mainly due to the production of reactive oxygen species by *Enterobacter* sp., which suppresses the formation of ookinetes [47].

#### 2.2.2. Inhibition of *Leishmania donovani*

Visceral leishmaniasis, commonly referred to as kala-azar or black fever, is a deadly protozoan disease resulting from *Leishmania donovani* and *Leishmania infantum* [48]. *Leishmania* possesses a digenetic life cycle: the extracellular, flagellated promastigote forms that live in the gut of the vectors, sand flies, and the obligate intracellular amastigote forms that colonize and proliferate within the phago-lysosomal system of mammalian macrophages [49]. Current treatment of leishmaniasis remains problematic, as available drugs are noxious and pricey and have bioavailability issues, and drug resistance has been found. Thus, there is an imminent demand for novel drugs to suppress these parasites [50]. Some pyridines, polyethers, macrotetrolide nactins, and macrolides, which are related to insect symbionts, exhibit great potential to suppress *L. donovani* [51,52] (Figure 3).

**Figure 4:**
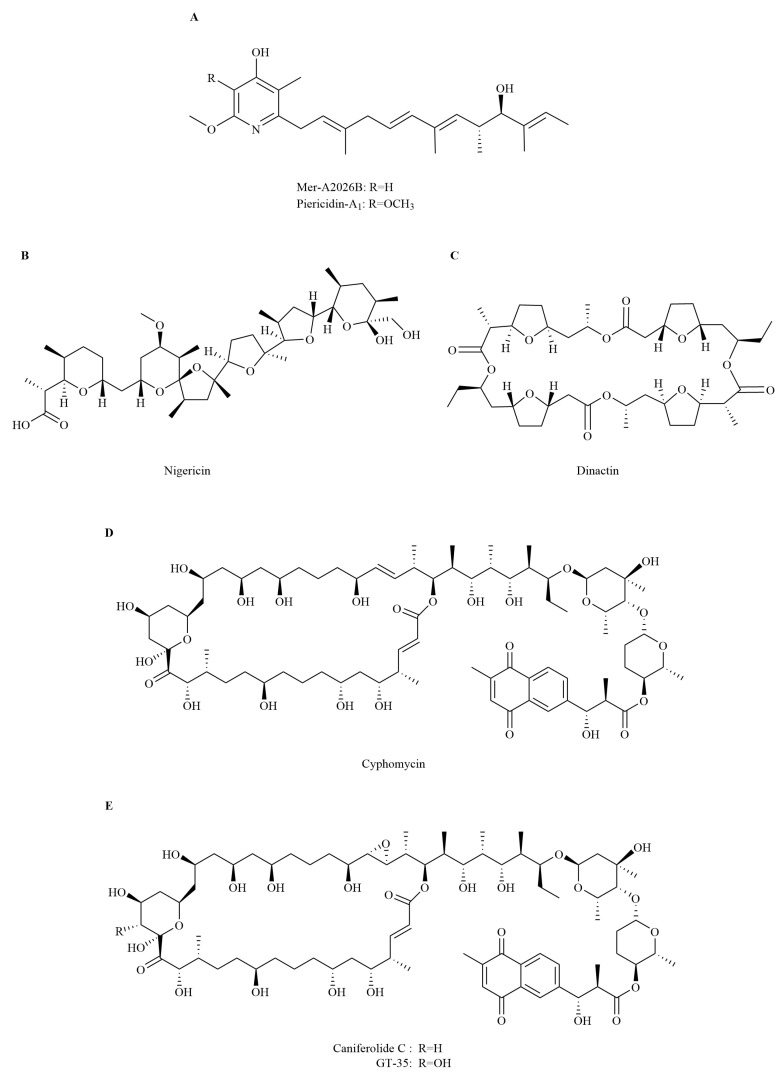
A selection of antiparasitic compounds isolated from insect symbionts. (**A**) Mer-A2026B and piericidin-A_1_; (**B**) Nigericin; (**C**) Dinactin; (**D**) Cyphomycin; (**E**) Caniferolide C and GT-35.

Pyridines Mer-A2026B and piericidin-A_1_, and the polyether nigericin (Figure 4A,B) produced by *Streptomyces* sp. ICBG292, separated from the exoskeleton of workers of attine ants *Cyphomyrmex* spp., exhibit inhibitory activity against intracellular amastigotes of *L. donovani* [51]. The close structural resemblance among Mer-A2026B, piericidin-A_1_, and coenzyme Q enables them to act as inhibitors of NADH-ubiquinone oxidoreductase in the mitochondrial electron transport chain, thereby exerting antibacterial capacity [53]. Nigericin can transport Na^+^ and K^+^ across membranes, thereby disrupting intracellular ion balance and adjusting pH [54,55]. *Leishmania* expels protons through H^+^-ATPase to adjust intracellular pH and, thus, to promote nutrient absorption. The influx of K^+^ into cells caused by nigericin increases the demand for proton expulsion and thereby increases the difficulty of nutrient absorption for *Leishmania*, thus suppressing the development of *Leishmania* [51].

*Streptomyces puniceus* ICBG378, separated from workers of the leaf-cutter ant *Acromyrmex rugosus rugosus*, generates dinactin that shows strong anti-*L. donovani* activity to combat promastigotes and intracellular amastigotes [51]. Dinactin (Figure 4C), a member of the macrotetrolide nactin family, is an ionophore that reversibly binds and transports ions across biomembranes. It is able to optionally bind to different kinds of cations, such as Na^+^, K^+^, NH_4_^+^, and Rb^+^ [51,56]. However, the subsequent physiological process of its *L. donovani*-inhibiting activity is unclarified. Dinactin is also known in other studies as a metabolite of actinomycetes with antibacterial and antitumor activity [57].

Three antifungal macrolides, viz., cyphomycin, caniferolide C, and GT-35 (Figure 4D,E), are separated from *Streptomyces* sp. ISID311, a symbiont related to *Cyphomyrmex* fungus-growing ants. These macrolides also show strong antiprotozoal activity against promastigotes and intracellular amastigotes of *L. donovani* [52]. The inhibitory activity of macrolides on *L. donovani* is attributed to their suppression of protein synthesis by targeting the cell ribosome, damaging cell membranes, and disrupting cellular physiological processes [58].

### 2.3. Inhibition of Tumor Cells

After the accumulation of gene mutations and chromosomal variations in normal cells reaches a threshold level, cell proliferation becomes out of control, resulting in the formation of tumor cells. The uncontrolled proliferation of tumor cells is primarily caused by the disruption of the cell cycle, particularly the mitotic process. The mitotic cell cycle consists of two phases, interphase and M phase. Abnormal cell cycle activity can arise from mutations in upstream signaling pathways or from genetic defects in genes that encode cell cycle proteins [59,60]. Moreover, cell death pathways, including apoptosis and anoikis, are downregulated by tumor cells to avoid programmed cell death. This is related to abnormal signaling pathways that control the mitochondrial pathway [61]. These pathways stabilize mitochondria and prevent cytochrome c from being released. If released, cytochrome c triggers the formation of apoptosome complexes, thereby inducing apoptosis [62].

Recent epidemiological data indicate that the cumulative risks of morbidity and mortality due to cancerous tumors are rising [63]. Currently, the approaches used to alleviate and treat cancer mainly include surgery, radiation therapy, chemotherapy, targeted drug therapy, immunotherapy, and personalized medicines. However, these approaches still come with drawbacks and side effects that may have an undesirable effect on the patient’s life. Therefore, it is an urgent demand to exploit novel nontoxic drug molecules for tumor therapy [64]. Tumor cell membranes are of high fluidity and abundant with negative charges and microvilli, which makes high selectivity of anti-tumor drugs toward tumor cells possible [26]. Insect symbionts are an important source of antitumor metabolites including peptides, polyketides, and other compounds. These compounds are promising to provide ideas for the development of nontoxic drugs.

#### 2.3.1. Peptides

**Figure 5:**
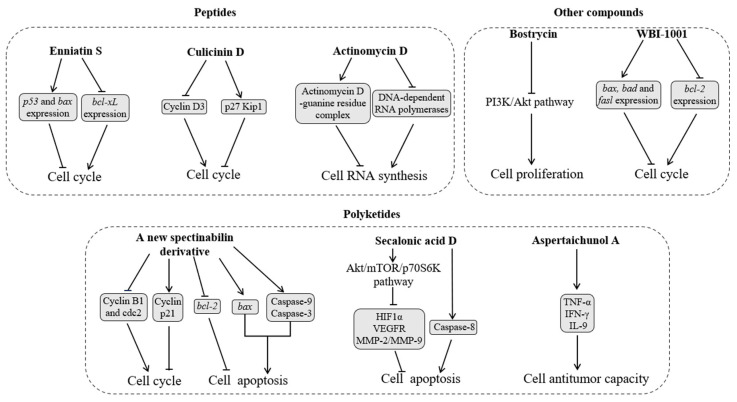
Effects of insect symbiont metabolites on tumor cells. Arrows depict activation, while bars represent suppression in molecular interactions. Abbreviations: Akt/mTOR/p70S6K—protein kinase B/mammalian target of rapamycin/p70 ribosomal protein S6 kinase; *bad*, *bax*, *fasl*, and *p53*—pro-apoptotic genes; *bcl-2* and *bcl-xL*—anti-apoptotic genes; Cyclin B1, cdc2, and D3—positive regulators of cell cycle progression; Cyclin p21—a negative regulator of cell cycle progression; HIF1α—the hypoxia-inducible factor 1α; IFN-γ—interferon gamma; IL-9—immunomodulatory factor interleukin-9; MMP-2/MMP-9—matrix metalloproteinase MMP-2/MMP-9; PI3K/Akt—phosphatidylinositol 3-kinase/protein kinase B; p27 Kip1—an inhibitor of the cell cycle; TNF-α—tumor necrosis factor alpha; VEGFR—vascular endothelial growth factor receptor.

**Figure 6:**
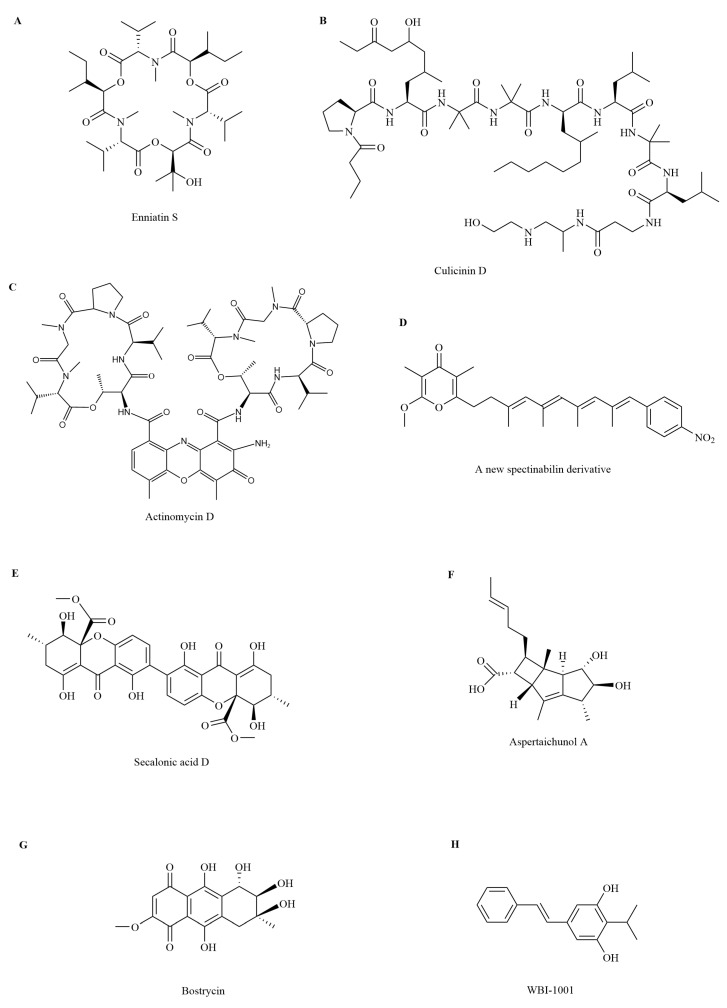
A selection of antitumor compounds isolated from insect symbionts. (**A**) Enniatin S; (**B**) Culicinin D; (**C**) Actinomycin D; (**D**) A new spectinabilin derivative; (**E**) Secalonic acid D; (**F**) Aspertaichunol A; (**G**) Bostrycin; (**H**) WBI-1001.

Peptides suppress tumor growth by regulating the cell division cycle and DNA transcription pathways (Figure 5). The cyclohexadepsipeptide enniatin S (Figure 6A) is separated from the solid culture of *Fusarium proliferatum*, a fungus derived from the debris of an unidentified insect in Tibet. Enniatin S upregulates the expression of pro-apoptotic genes *p53* and *bax* and downregulates the expression of the anti-apoptotic *bcl-2* family member *bcl-xL*. It also induces human chronic myeloid leukemia cell line K562 arrest in the G_0_/G_1_ phase of the cell division cycle. This cell cycle arrest is helpful in suppressing the proliferation of tumor cells [65,66].

Culicinin D (Figure 6B) generated by *Culicinomyces clavisporus* LL-12I252 separated from the gut of *Forcipomyia marksae* larvae suppresses cyclin D3, a positive regulator of cell cycle progression, and raises the level of p27 Kip1, an inhibitor of the cell cycle. Overall, the inhibitory activity of culicinin D on tumor cell growth might be achieved by inhibiting the mTOR signaling pathway. Culicinin D exhibits strong but selective suppression against the breast tumor cell line MDA468 [67].

Actinomycin D (Figure 6C) generated by *Streptomyces* sp. Av25_2, which is separated from the fungus gardens of the ant *A. octospinosus*, is an antitumor agent that interrupts tumor cell RNA synthesis by binding to guanine residues and suppressing DNA-dependent RNA polymerases [68,69].

#### 2.3.2. Polyketides

Polyketides exhibit antitumor activity by regulating cell physiological functions, such as the division cycle, cell apoptosis, and T-cell function (Figure 3). A new spectinabilin derivative (A) (Figure 6D) has been separated from the culture media of the ant-derived *Streptomyces* sp. 1H-GS5 [10]. Compound A demonstrates toxicity against human hepatocellular carcinoma cell lines SMMC7721 and HepG2 through two mechanisms [70]: (1) it arrests SMMC7721 and HepG2 cells in the G2/M phase cell cycle by lowering the protein levels of cyclin B1 and cdc2 and raising that of p21; and (2) it downregulates the expression of *bcl*-*2*, upregulates the expression of *bax*, and triggers the cleavage of caspase-9 and -3, thus activating apoptosis in SMMC7721 and HepG2 cells.

*Penicillium chrysogenum* separated from the gut of the true bug *Aspongopus chinensis* generates secalonic acid D (Figure 6E), which demonstrates strong antiangiogenic antitumor activity [71]. Secalonic acid D suppresses tumor cell survival by targeting the Akt/mTOR/p70S6K pathway, which, in turn, affects key proangiogenesis factors, such as the hypoxia-inducible factor 1α, vascular endothelial growth factor receptors, and the matrix metalloproteinase MMP-2/MMP-9. Apoptosis induced by secalonic acid D starts with the disruption of mitochondrial membrane potential, which correlates with a significant reduction in the *bcl-2*/*bax* ratio. The *bcl-2*/*bax* ratio acts as a key regulator of the antioxidant pathway and cell death. Meanwhile, secalonic acid D treatment also upregulates the expression of caspase-8, which is a key factor in cell apoptosis and the extrinsic apoptosis pathway. These results suggest that secalonic acid D triggers tumor cell apoptosis through both extrinsic and intrinsic mechanisms [72].

Aspertaichunol A (Figure 6F), a polyketide with a novel scaffold, is separated from *Aspergillus taichungensis* SMU01, an endosymbiont of the American cockroach *Periplaneta americana*. Aspertaichunol A increases the expression and secretion of immunomodulatory factors interleukin-9, interferon gamma (IFN-γ), and tumor necrosis factor alpha (TNF-α) in Th9 cells. TNF-α synergistically recruits and activates macrophages with IFN-γ to boost the antitumor activity of Th9 cells, facilitating the defensive response of the host [73].

#### 2.3.3. Other Compounds

Insect symbionts also generate other compounds that exhibit antitumor activity by triggering cell apoptosis and downregulating the PI3K/Akt pathway (Figure 5). Fatty acids generated by *Mucor bainieri* MK-Bee-2 separated from *A. mellifera* trigger apoptosis in cancer cell lines human lung carcinoma A549 and hepatocellular carcinoma HepG2 [74]. *Streptomyces* sp. strain ess_amH1, separated from the gut of *A. mellifera yemintica*, generates olefins, alkanes, and esters, which demonstrate in vitro anticancer activity against cell lines breast cancer MCF7 and hepatocarcinoma HepG2 [75]. The entomopathogenic fungus *Aspergillus* sp. generates an anthraquinone bostrycin (Figure 6G), which suppresses the multiplication of human lung carcinoma A549 cells by downregulating the PI3K/Akt pathway [76,77]. The styrene derivative WBI-1001 (Figure 6H) separated from an insect symbiont *Xenorhabdus* sp. downregulates the expression of the gene *bcl-2* mRNA; upregulates the expression of pro-apoptotic genes *bax*, *bad*, and *fasl* mRNA; and arrests A549 cells in the G1 phase [78,79].

## 3. Medical Application of Insect Symbionts

### 3.1. Status Quo and Existing Problems

#### 3.1.1. Application of Symbionts

Insect symbionts hold promising application prospects in the medical field due to their ability to treat human diseases. For instance, probiotic lactobacilli isolated from *B. mori*, such as *Lactobacillus acidophilus*, *Lactobacillus paracasei*, *Lactobacillus plantarum*, and *Lactobacillus rhamnosus*, can be formulated as oral suspensions and nasal sprays and used to eradicate the persistent carriage of meticillin-resistant *S. aureus* [80,81]. Lactic acid bacteria isolated from the stomach of *A. mellifera* can treat wounds infected with *S. aureus*, *P*. *aeruginosa*, and vancomycin-resistant *Enterococcus*. Formic acid and lactic acid produced by lactic acid bacteria can lower the pH of the wound environment and inhibit the growth of bacteria and may help inhibit wound deterioration [82]. Lactic acid bacteria also produce hydrogen peroxide, CO_2_, diacetyl, and bacteriocins, which may create an unfavorable environment for the growth of pathogenic microorganisms and interact synergistically with fatty acids in wound healing [83].

The application of insect symbionts in the medical field still faces numerous challenges: (1) it is difficult to culture insect symbionts in vitro, as most of the insect bacterial symbionts are unable to survive on artificial media [12]; (2) it is very difficult for ingested probiotic isolates to reach target sites in the human body [14]; (3) research results related to the medical efficacy of some symbionts are inconsistent, finding them effective in some studies while ineffective in other studies [84,85]; and (4) probiotics can cause systemic infections, harmful metabolic activities, excessive immune stimulation in susceptible individuals, and gene transfer within symbionts and even between symbionts and pathogens [13,86].

On rare occasions, probiotics may translocate from the gastrointestinal tract to the hemolymph, resulting in invasive infections, which are associated with sepsis, bacteremia, and fungemia [87]. In immunocompromised states, abnormal metabolic activity in probiotics (especially *Lactobacillus* and *Streptococcus* sp.) can cause D-lactic acidosis, which may lead to brain fogginess [88]. Children who ingest probiotics (*Lactobacillus reuteri* and *L. rhamnosus* GG) through the intake of probiotic food supplements in the first year of life have a slightly increased risk of celiac disease autoimmunity [89]. The survivability of pathogens against the exposure and administration of antibiotics that could kill them or restrict their proliferation is called antibiotic resistance [90]. The risk of horizontal transfer of antibiotic resistance genes from probiotics to potential pathogens in the gut is supported by the identification of pAM*β*1 plasmid, related to macrolide lincosamide and streptogrammin B resistance and derived from probiotic *L. reuteri*, in mouse fecal isolates of *E. faecalis* [87,91].

#### 3.1.2. Application of Symbiont Metabolites

Insect symbionts produce complex metabolites with diverse functions to inhibit human pathogenic bacteria and fungi, parasites, and tumors, some of which are expected to be used to treat human diseases. For example, *Bacillus* sp. in the eggs of the brown planthopper *Nilaparavata lugens* produces polymyxins [92]. In the clinical setting, polymyxins are used to treat multiple-drug-resistant infections and meningitis caused by Gram-negative pathogenic bacteria through intraventricular and intravenous instillation, and they are also used as an aerosol to treat lung infections caused by bronchitis [93]. WBI-1001, produced by insect-symbiotic bacteria *Xenorhabdus* sp., is effective for the treatment of mild to moderate psoriasis [78,94,95]. In addition, exopolysaccharides produced by lactic acid bacteria have nontoxic properties and good biocompatibility. They have the potential as drug carriers to alleviate drug stimulation and immune response to human tissues and have the function of gene delivery, which can treat cancer genes. Exopolysaccharide-based nanocarriers have been used in various medical diagnostic and bioimaging applications. However, practical applications still need further research and clinical trial development [96].

Most symbiont metabolites are still in a preliminary research stage, and their medical application faces many challenges: (1) there is resistance to symbiont metabolites [97]; and (2) the application of medical metabolites for clinical treatment may also cause some adverse reactions [94]. An adverse reaction to a drug has been defined as any noxious or unintended reaction to a drug that is administered in standard doses by the proper route for the purpose of prophylaxis, diagnosis, or treatment [98]. For example, adverse events of WBI-1001 are target site reactions such as hyperpigmentation, dermatitis, folliculitis, and papules [94]. These issues faced in the medical application of symbiont metabolites are not different from those of new drugs derived from other sources. The unique problem associated with the application of symbiont metabolites is that insect symbionts often require specific environmental conditions to generate the metabolites of interest. Some symbionts only produce metabolites within insect partners and not in vitro. Some symbionts have silenced metabolite genes, and only a small portion of active metabolites can be obtained through conventional methods [99].

### 3.2. Possible Solutions

#### 3.2.1. Application of Insect Cell Cultures

The reason behind the difficulty in culturing insect symbionts in vitro is that the genome of insect symbionts is small and lacks the gene sets for independent replication. Consequently, only a small portion of insect symbionts are culturable in common culture media. To overcome this limitation, insect cell cultures are raised to release growth factors into the medium before endosymbionts are cultured [12]. For example, the aphid symbionts (*Candidatus Consessoris aphidicola* and *Candidatus Adiaceo aphidicola*) are successfully cultured with insect cell lines [100]. In addition, insect symbionts can be cultured in vitro through the organ culture method, which has been used to cultivate *Burkholderia* spp. that inhabit the crypts of the Southern chinch bug *Blissus insularis*. The *B. insularis* crypts incubated in osmotically balanced insect cell culture media enable *Burkholderia* spp. to make the transition to an in vitro environment and to be subsequently cultured in standard bacteriological media [101].

#### 3.2.2. Screening of New Symbiont Stains and Metabolites

There is a need to screen more symbionts and assess their medical potential and safety. The development of high-throughput sequencing technologies facilitates the screening of symbionts, such as the identification of potential probiotics (*L. acidophilus*, *L. plantarum*, *L. paracasei*, and *L. rhamnosus*) in the midgut of *B. mori* through a metagenomic approach [81]. Whole-genome sequencing enables the identification of genes for virulence, toxins, and antibiotic resistance, as well as the clear assignment of species and strain identity. For example, assessing the risk of antibiotic resistance gene transfer within probiotic genomes requires whole-genome sequencing and the identification of antibiotic resistance genes from the sequence. If the resistance gene is for an antibiotic that is not deemed clinically relevant, it poses a low risk and would be considered for use in the medical field [87].

The use of genomics tools broadens the screening range of metabolites in symbionts. The compound synthesis genes that usually participate in the molecular recognition of the cellular target, such as the sugar synthesis gene, can be screened using genomics tools. For example, sipanmycins A and B with potential implications in cancer chemotherapy are identified from metabolites of *Streptomyces*, isolated from the integument of *Attini* ants, by screening genes that participate in the biosynthesis of 6-deoxyhexoses [102].

#### 3.2.3. Combination of Multiple Strains

When multiple symbiont strains are used together, the exchange of different metabolites between different strains could enhance the chance of their survival and colonization in different niches and improve their application efficacy. For example, the adhesion to the human intestinal mucus of *Propionibacterium freudenreuchii* P6 is more than tripled by the presence of *L. rhamnosus* GG [103]. The feature of the stimulation of the adhesion of one strain by another greatly enhances the successful colonization of multi-strain probiotics, which enables probiotics to inhibit and prevent the colonization of pathogens [104,105]. Three *Lactobacillus* strains (*L. acidophilus* B21190, *L. paracasei* B21060, and *L. paracasei* B21070) have been tested for their individual and combined activity against selected enteropathogens (*E. coli*, *Salmonella enteritidis*, and *Vibrio cholerae*). Only the mixture of three *Lactobacillus* strains is able to almost completely inhibit the growth of *E. coli* and *S. enteritidis* [106]. A multi-strain preparation containing three different *Lactobacillus* strains, namely, *L. acidophilus*, *L. bifidus*, and *L. rhamnosus*, and probiotics containing multiple species of lactobacilli and bifidobacteria are more effective in preventing dysbiosis induced by ceftriaxone treatment than other probiotic preparations [104].

#### 3.2.4. Combination of Symbionts with Synergists

Another solution to improve the application efficacy of symbionts is to combine symbionts with synergistic components. Phenolic compounds, commonly present in fruit and vegetables, are used simultaneously with *L. acidophilus* and *L. rhamnosus*. Phenolic compounds selectively inhibit the growth of pathogenic bacteria (*E. coli* and *S. typhimurium*) without affecting the viability of probiotics [107]. Furthermore, probiotics may use phenolic compounds as substrates to increase their survival and functionality. Studies have shown that phenolic compounds can improve the adhesion capacity and survival of probiotics during exposure to conditions that mimic the gastrointestinal tract [108]. In addition, anthocyanins extracted from purple sweet potatoes induce the growth of *Bifidobacterium* spp., *Enterococcus*, and *Lactobacillus* but inhibit the growth of *Bacteroides*-*Prevotella* spp. and *Clostridium histolyticum*. The combination of probiotics and anthocyanins enhances the antibacterial effect of symbionts [109], which can contribute to the formulation of nutraceutical products that improve the overall health of humans [107].

#### 3.2.5. Adverse Reaction Surveillance and Prediction

The antibiotic resistance and adverse reactions caused by insect symbionts or their metabolites hinder their application in the medical field [94,97]. Antibiotic resistance surveillance systems provide valuable data that are helpful for understanding and predicting trends in resistance and developing, implementing, and monitoring new empirical antibiotic prescribing [110]. Therapeutic drug monitoring helps to solve the problem of adverse reactions in clinical practice. Therapeutic drug monitoring involves collecting blood samples from patients, using analytical techniques to determine drug concentrations, and adjusting dosing regimens based on pharmacokinetic principles to achieve personalized treatment. Therapeutic drug monitoring may increase the probability of a successful outcome, prevent drug-related toxicity, and potentially prevent the emergence of antibiotic resistance [111].

Further genomic developments will allow the development of predictive tests for the actions of drugs, including adverse drug reactions, enabling more tailored therapy for the individual [112]. For example, research has shown that individuals carrying the HLA-B*58:01 gene have a significantly increased risk of developing severe allergic reactions after using allopurinol. Therefore, performing HLA-B*58:01 gene testing on patients before using allopurinol can help predict allergy risk and avoid the use of the drug in high-risk patients [113].

## 4. Summary and Outlook

Insect symbionts have the potential to solve the problems of drug resistance of human pathogenic bacteria and fungi and of human parasites and tumors. Fatty acids, antibacterial peptides, polyene macrolides, alkaloids, and roseoflavin produced by some insect symbionts inhibit human pathogenic bacteria and fungi. Lipases, yeast killer toxins, reactive oxygen species, pyridines, polyethers, macrotetrolide nactins, and macrolides have the ability to kill parasites. Peptides and polyketides inhibit human tumors. Although some insect symbionts have been used in clinical research with significant achievements, there still remain substantial challenges for their application, such as difficulty in the culture of insect symbionts in vitro, difficulty in targeting bacteria to specific sites in the human body, the limited capability of symbionts to produce active metabolites in vitro, inconsistent clinical research results of symbionts, adverse reactions on humans, and the development of antibiotic resistance. These challenges can be overcome by the application of insect cell cultures, the screening of new symbiont strains and metabolites, the combination of multiple symbiotic bacteria, the combination of symbiotic bacteria with synergists, and adverse reaction surveillance and prediction.

The development of high-throughput screening technology, directed evolution, and metabolic engineering enables comprehensive investigations into insect symbionts and their metabolites. For example, a biosensor based on anthranilate synthase component I promoter pA can respond to L-lysine signals in *E. coli* and transfer them to signals that can be detected by the fluorescence-activated cell sorter. The high-throughput screening method combining the biosensor and the fluorescence-activated cell sorter has been used to identify a high-producing strain of *E. coli* for L-lysine production [114]. Directed evolution—a laboratory process in which biological entities with desired traits are created through site-directed mutagenesis, iterative rounds of genetic diversification, and library screening or selection—has been used to improve the productivity and saccharification efficiency of the cellulolytic β-glucosidase in *Pichia pastoris* [115,116]. Metabolic engineering, which involves the enhancement of cellular activities through the manipulation of enzymatic, transport, and regulatory functions of the cell using recombinant DNA technology, has led to an increase in mono-rhamnolipid production when *E. coli* is used as a heterologous host [117,118].

## Data Availability

No new data were created or analyzed in this study.

## Supplementary Materials

The following supporting information can be downloaded at: https://www.mdpi.com/article/10.3390/insects16050457/s1, Table S1: Insect symbionts that produce antibacterial metabolites. References [8,16,17,22,23,28,32,33,38,39,40,71,119,120,121,122,123,124,125,126,127,128,129,130,131,132,133,134,135,136,137,138,139,140,141] are cited in the Supplementary Materials.
