# Supplementary material for: Medical Potential of Insect Symbionts
Source: Insects. 2025 Apr 26;16(5):457. doi: 10.3390/insects16050457 (PMC12111880; doi:10.3390/insects16050457)
Supplement: Supplementary file 1 [file insects-16-00457-s001.zip › insects-3566400-supplementary.pdf]

Table S1 Insect symbionts that produce antibacterial metabolites

Insect host	Parasitism site	Symbiont	Antibacterial activity	Reference
Acromyrmex octospinosus	Body surface, fungus garden	*Streptomyces* spp.	Candicidin D produced by symbionts inhibits the growth of *Escovopsis weberi*.	[1]
Allomerus	Cuticle	*Amycolatopsis* sp. FG22, *Streptomyces* sp. FG23, FG25, and FG26	Symbionts inhibit the growth of *Candida albicans*.	[2]
Apis mellifera	Gut	*Lactobacillus casei*	The symbiont inhibits the growth of *Escherichia coli*, *Salmonella typhimurium*, and *Staphylococcus aureus* by producing lactic, oxalic, glutaric and acetic acids.	[3]
	Stomach	Lactic acid bacteria PAM3 and PAM4	The symbiont inhibits the growth of *Staphylococcus aureus* and *Salmonella typhimurium* by producing fatty acids.	[4]
	Honey	*Lactobacillus* sp.	The symbiont inhibits the growth of *Listeria monocytogenes* and *Escherichia coli* by producing acetic and lactic acids.	[5]
Apterostigma	Ant nests	*Pseudomonas* sp.	Selvamicin produced by the symbiont interacts with ergosterol on the cell membrane of *Candida albicans*, thus compromising the integrity of the membrane and inhibiting the function of membrane proteins.	[6]

Aspongopus chinensis	Gut	*Penicillium chrysogenum*	The symbiont inhibits the growth of *Staphylococcus aureus* by producing emodin.	[7]
Copris tripartitus	Gut	*Streptomyces* sp. SNU607	Coprisidins A and B produced by the symbiont inhibit the growth of *Staphylococcus aureus*.	[8]
Drosophila melanogaster	Gut	*Cryptococcus albidus, Kluyveromyces lactis, Metschnikowia pulcherrima, Pichia toletana,* Saccharomyces *cerevisiae,* and *Rhodotorula mucilaginosa*	The symbionts inhibit the growth of *Aspergillus nidulans* by producing as yet unknown metabolites.	[9]
Hermetia illucens	Gut	*Bacillus* spp.	The symbionts inhibit the growth of *Staphylococcus aureus* by producing lipopeptides.	[10]
Heterotrigona itama	Honey	Lactic acid bacteria Sy-1, Sy-2, Sy-3, and Sy-4	Bacteriocins produced by the symbionts inhibit the growth of *Bacillus subtilis, Escherichia coli, Klebsiella pneumoniae, Pseudomonas aeruginosa,* and *Staphylococcus aureus* by interfering with the formation of biofilms of pathogenic bacteria.	[11]
Locusta migratoria	Gut	*Amycolatopsis* sp. HCa4	The symbiont inhibits the growth of methicillin-resistant *Staphylococcus aureus* by producing two new macrolactams.	[12]
Macrotermes barneyi	Fungus garden	*Streptomyces* strain HF10	Pentamycin produced by the symbiont is a	[13,14]

			polyene macrolide with a broad spectrum of antimicrobial activity.	
Macrotermes natalensis	Gut	*Amycolatopsis* sp. M39	The symbiont inhibits the growth of *Staphylococcus aureus* by producing macrotermycins A–D.	[15]
	Fungus comb, abdomen	*Bacillus* spp. #9, and #11	Bacillaene A produced by the symbionts displays antibacterial activity against *Bacillus thuringiensis, Escherichia coli, Klebsiella pneumoniae, Proteus vulgaris, Serratia marcescens*, and *Staphylococcus aureus*.	[16]
Melipona scutellaris	Entire body	*Streptomyces* sp. ICBG1323, and *Micromonospora* sp. ICBG1321	Lobophorin B produced by the symbionts shows antibacterial activities against *Staphylococcus aureus* ATCC 29213 and *Enterococcus faecalis* ATCC 29212.	[17,18]
Microtermes sp.	Gut	*Pseudoxylaria* sp. X802	The symbiont inhibits the growth of *Pseudomonas aeruginosa* by producing pseudoxylallemycins A–D.	[19]
Nicrophorus concolor	Gut	*Mycobacterium*	The symbionts inhibit the growth of *Staphylococcus aureus* and *Enterococcus faecalis* by producing nicrophorusamide A.	[20,21]
Nicrophorus vespilloides	Gut	*Serratia marcescens*	The symbiont inhibits the growth of	[22,23]

			methicillin-resistant *Staphylococcus aureus* and *Listeria monocytogenes* by producing serrawettin W2.	
Odontotermes formosanus	Body surface	*Streptomyces davaonensis*	Roseoflavin produced by the symbiont inhibits the synthesis of bacterial flavin mononucleotide and thus inhibits the synthesis of bacterial riboflavin.	[24,25]
	Gut	*Streptomyces koyangensis*	Indolizine produced by the symbiont inhibits the dihydrofolate reductase and thus interferes with tetrahydrofolate metabolism of *Staphylococcus aureus* and *Candida albicans*.	[26,27]
Onthophagus lenzii	Oral excretion	*Brevibacillus* sp. PTH23	Lenzimycins A and B produced by the symbiont inhibit the growth of *Bacillus* sp. CCARM 9248, *Enterococcus faecium*, and certain strains of *Enterococcus faecalis*.	[28]
Philanthus triangulum	Cocoon	*Candidatus Streptomyces philanthi*	Streptochlorin produced by the symbiont has broad-spectrum antibacterial activity.	[26]
Polistes dominula	Nest	*Actinoplanes*, *Micromonospora*, and *Streptomyces*	Antimicrobials produced by the symbionts inhibit the	[29]

			growth of *Bacillus subtilis*, *Escherichia coli*, *Pseudomonas aeruginosa*, *Serratia marcescens*, and *Staphylococcus aureus*.	
Riptortus pedestris	Gut	*Nodulisporium* sp. IFB-A163	The symbiont inhibits the growth of *Pseudomonas aeruginosa* by producing nodupetides.	[30]
Sceliphron caementarium	Gut	*Streptomyces* sp.	The symbiont inhibits the growth of *Candida albicans* by producing sceliphrolactam.	[31]
Spodoptera littoralis	Gut	*Enterococcus mundtii*	The symbiont inhibits the growth of *Enterococcus casseliflavus* by producing mundticin KS.	[32]
Trachymyrmex septentrionalis	Laterocervical plates	*Pseudonocardia* sp.	Thiopeptide GE37468 produced by the symbiont inhibits the growth of methicillin-resistant *Staphylococcus auereus*.	[33,34]
Trachymyrmex sp.	Nest	*Pseudonocardia* sp.	Conocandins B and C produced by the symbiont inhibit the growth of *Escovopsis* sp.	[35]

References

1. Haeder, S.; Wirth, R.; Herz, H.; Spiteller, D. Candicidin-producing *Streptomyces* support leaf-cutting ants to protect their fungus garden against the pathogenic fungus *Escovopsis*. *Proc. Natl. Acad. Sci. U. S. A.* **2009**, *106*, 4742–4746. doi: 10.1073/pnas.0812082106
2. Seipke, R.F.; Barke, J.; Ruiz-Gonzalez, M.X.; Orivel, J.; Yu, D.W.; Hutchings, M.I. Fungus-growing *Allomerus* ants are associated with antibiotic-producing actinobacteria. *Antonie Van Leeuwenhoek* **2012**, *101*, 443–447. doi:10.1007/s10482-011-9621-y
3. Elzeini, H.M.; Ali, A.R.A.A.; Nasr, N.F.; Elenany, Y.E.; Hassan, A.A.M. Isolation and identification of lactic acid bacteria from the intestinal tracts of honey bees, *Apis mellifera* L., in Egypt. *J. Apic. Res.* **2021**, *60*, 349–357. doi:10.1080/00218839.2020.1746019

4. Nadia, A.B.; Jannah, S.N.; Purwantisari, S. Isolation and characterization of lactic acid bacteria from *Apis mellifera* stomach and their potential as antibacterial using *in vitro* test against growth of *Staphylococcus aureus* and *Salmonella typhimurium*. *NICHE J. Trop. Biol.* **2020**, *3*, 35–44. doi:10.14710/niche.3.1.35-44
5. Putri, I.; Jannah, S.N.; Purwantisari, S. Isolation and characterization of lactic acid bacteria from *Apis mellifera* and their potential as antibacterial using *in vitro* test against growth of *Listeria monocytogenes* and *Escherichia coli*. *NICHE J. Trop. Biol.* **2020**, *3*, 26–34. doi:10.14710/niche.3.1.26-34
6. Van Arnam, E.B.; Ruzzini, A.C.; Sit, C.S.; Horn, H.; Pinto-Tomas, A.A.; Currie, C.R.; Clardy, J. Selvamicin, an atypical antifungal polyene from two alternative genomic contexts. *Proc. Natl. Acad. Sci. U. S. A.* **2016**, *113*, 12940–12945. doi:10.1073/pnas.1613285113
7. Li, T.X.; Su; H.Y.; Yu, J.C.; Hao, H.; Jia, X.W.; Shi, F.C.; Xu, C.P. Antibacterial metabolites from the beetle-associated fungus *Penicillium chrysogenum*. *An. Acad. Bras. Cienc.* **2023**, *95*, e20220178. doi:10.1590/0001-3765202320220178
8. Um, S.; Bach, D.H.; Shin, B.; Ahn, C.H.; Kim, S.H.; Bang, H.S.; Oh, K.B; Lee, S.K; Shin, J.; Oh, D.C. Naphthoquinone–oxindole alkaloids, coprisidins A and B, from a gut-associated bacterium in the dung beetle, *Copris tripartitus*. *Org. Lett.* **2016**, *18*, 5792–5795. doi:10.1021/acs.orglett.6b02555
9. Rohlfs, M.; Kurschner, L. Saprophagous insect larvae, *Drosophila melanogaster*, profit from increased species richness in beneficial microbes. *J. Appl. Entomol.* **2010**, *134*, 667–671. doi:10.1111/j.1439-0418.2009.01458.x
10. Zhang, Y.; Xiao, X.; Elhag, O.; Cai, M.; Zheng, L.; Huang, F.; Jordan, H.R.; Tomberlin, J.K.; Sze, S.H.; Yu, Z.N.; et al. *Hermetia illucens* L. larvae-associated intestinal microbes reduce the transmission risk of zoonotic pathogens in pig manure. *Microb. Biotechnol.* **2022**, *15*, 2631–2644. doi:10.1111/1751-7915.14113
11. Syed Yaacob, S.N.; Huyop, F.; Kamarulzaman Raja Ibrahim, R.; Wahab, R.A. Identification of *Lactobacillus* spp. and *Fructobacillus* spp. isolated from fresh *Heterotrigona itama* honey and their antagonistic activities against clinical pathogenic bacteria. *J. Apic. Res.* **2018**, *57*, 395–405. doi:10.1080/00218839.2018.1428047
12. Xiao, Y.S.; Zhang, B.; Zhang, M.; Guo, Z.K.; Deng, X.Z.; Shi, J.; Li, W.; Jiao, R.H.; Tan, R.X.; Ge, H.M. Rifamorpholines A–E, potential antibiotics from locust-associated actinobacteria *Amycolatopsis* sp. Hca4. *Org. Biomol. Chem.* **2017**, *15*, 3909–3916. doi:10.1039/c7ob00614d
13. Li, J.J.; Sang, M.L.; Jiang, Y.T.; Wei, J.H.; Shen, Y.L.; Huang, Q.H.; Li, Y.Y.; Ni, J.F. Polyene-producing *Streptomyces* spp. from the fungus-growing termite *Macrotermes barneyi* exhibit high inhibitory activity against the antagonistic fungus *Xylaria*. *Front. Microbiol.* **2021**, *12*, 649962. doi:10.3389/fmicb.2021.649962
14. Frey Tirri, B.; Bitzer, J.; Geudelin, B.; Drewe, J. Safety, tolerability and pharmacokinetics of intravaginal pentamycin. *Chemother.* **2010**, *56*, 190–196. doi:10.1159/000316329
15. Beemelmanns, C.; Ramadhar, T.R.; Kim, K.H.; Klassen, J.L.; Cao, S.; Wyche, T.P.; Hou, Y.P.; Poulsen, M.; Bugni, T.S.; Currie, C.R.; et al. Macrotermycins A-D, glycosylated macrolactams from a termite-associated *Amycolatopsis* sp. M39. *Org. Lett.* **2017**, *19*, 1000–1003. doi:10.1021/acs.orglett.6b03831
16. Um, S.; Fraimout, A.; Sapountzis, P.; Oh, D.C.; Poulsen, M. The fungus-growing termite *Macrotermes natalensis* harbors bacillaene-producing *Bacillus* sp. that inhibit potentially antagonistic fungi. *Sci. Rep.* **2013**, *3*, 3250. doi:10.1038/srep03250
17. Rodriguez-Hernandez, D.; Melo, W.G.; Menegatti, C.; Lourenzon, V.B.; do Nascimento, F.S.; Pupo, M.T. Actinobacteria associated with stingless bees biosynthesize bioactive polyketides against bacterial pathogens. *New J. Chem.* **2019**, *43*, 10109–10117. doi:10.1039/c9nj01619h
18. Niu, S.W.; Li, S.M.; Chen, Y.C.; Tian, X.P.; Zhang, H.B.; Zhang, G.T.; Zhang, W.M.; Yang, X.H.; Zhang,

S.; Ju, J.H.; et al. Lobophorins E and F, new spirotetronate antibiotics from a South China Sea-derived *Streptomyces* sp. SCSIO 01127. *J. Antibiot.* **2011**, *64*, 711–716. doi:10.1038/ja.2011.78

19. Guo, H.; Kreuzenbeck, N.B.; Otani, S.; Garcia-Altares, M.; Dahse, H.M.; Weigel, C.; Aanen, D.K.; Hertweck, C.; Poulsen, M.; Beemelmanns, C. Pseudoxylallemycins A-F, cyclic tetrapeptides with rare allenyl modifications isolated from *Pseudoxylaria* sp. X802: A competitor of fungus-growing termite cultivars. *Org. Lett.* **2016**, *18*, 3338–3341. doi:10.1021/acs.orglett.6b01437
20. Shin, Y.H.; Bae, S.; Sim, J.; Hur, J.; Jo, S.I.; Shin, J.; Suh, Y.G.; Oh, K.B.; Oh, D.C. Nicrophorusamides A and B, antibacterial chlorinated cyclic peptides from a gut bacterium of the carrion beetle *Nicrophorus concolor*. *J. Nat. Prod.* **2017**, *80*, 2962–2968. doi:10.1021/acs.jnatprod.7b00506
21. Jang, S.; Kikuchi, Y. Impact of the insect gut microbiota on ecology, evolution, and industry. *Curr. Opin. Insect Sci.* **2020**, *41*, 33–39. doi:10.1016/j.cois.2020.06.004
22. Heise, P.; Liu, Y.; Degenkolb, T.; Vogel, H.; Schaberle, T.F.; Vilcinskas, A. Antibiotic-producing beneficial bacteria in the gut of the burying beetle *Nicrophorus vespilloides*. *Front. Microbiol.* **2019**, *10*, 1178. doi:10.3389/fmicb.2019.01178
23. Lee, E.R.; Blount, K.F.; Breaker, R.R. Roseoflavin is a natural antibacterial compound that binds to FMN riboswitches and regulates gene expression. *RNA Biol.* **2009**, *6*, 187–194. doi:10.4161/rna.6.2.7727
24. Zhou, L.F.; Wu, J.; Li, S.; Li, Q.; Jin, L.P.; Yin, C.P.; Zhang, Y.L. Antibacterial potential of termite-associated *Streptomyces* spp. *ACS Omega* **2021**, *6*, 4329–4334. doi:10.1021/acsomega.0c05580
25. Rao, K.N.; Venkatachalam, S.R. Inhibition of dihydrofolate reductase and cell growth activity by the phenanthroindolizidine alkaloids pergularinine and tylophorinidine: The *in vitro* cytotoxicity of these plant alkaloids and their potential as antimicrobial and anticancer agents. *Toxicol. in vitro* **2000**, *14*, 53–59. doi:10.1016/S0887-2333(99)00092-2
26. Bi, S.F.; Guo, Z.K.; Jiang, N.; Jiao, R.H.; Ge, H.M.; Tan, R.X. New alkaloid from *Streptomyces koyangensis* residing in *Odontotermes formosanus*. *J. Asian Nat. Prod. Res.* **2013**, *15*, 422–425. doi:10.1080/10286020.2013.767246
27. An, J.S.; Hong, S.H.; Somers, E.; Lee, J.; Kim, B.Y.; Woo, D.; Kim, S.W.; Hong, H.J.; Jo, S.I.; Shin, J.; et al. Lenzimycins A and B, metabolites with antibacterial properties from *Brevibacillus* sp. associated with the dung beetle *Onthophagus lenzii*. *Front. Microbiol.* **2020**, *11*, 599911. doi:10.3389/fmicb.2020.599911
28. Koehler, S.; Doubsky, J.; Kaltenpoth, M. Dynamics of symbiont-mediated antibiotic production reveal efficient long-term protection for beewolf offspring. *Front. Zool.* **2013**, *10*, 3. doi:10.1186/1742-9994-10-3
29. Madden, A.A.; Grassetti, A.; Soriano, J.A.N.; Starks, P.T. Actinomycetes with antimicrobial activity isolated from paper wasp (Hymenoptera: Vespidae: Polistinae) nests. *Environ. Entomol.* **2013**, *42*, 703–710. doi:10.1603/EN12159
30. Wu, H.M.; Lin, L.P.; Xu, Q.L.; Han, W.B.; Zhang, S.; Liu, Z.W.; Mei, Y.N.; Yao, Z.J.; Tan, R.X. Nodupetide, a potent insecticide and antimicrobial from *Nodulisporium* sp. associated with *Riptortus pedestris*. *Tetrahedron Lett.* **2017**, *58*, 663–665. doi:10.1016/j.tetlet.2017.01.009
31. Oh, D.C.; Poulsen, M.; Currie, C.R.; Clardy, J. Sceliphrolactam, a polyene macrocyclic lactam from a wasp-associated *Streptomyces* sp. *Org. Lett.* **2011**, *13*, 752–755. doi:10.1021/ol102991d
32. Shao, Y.; Chen, B.; Sun, C.; Ishida, K.; Hertweck, C.; Boland, W. Symbiont-derived antimicrobials contribute to the control of the lepidopteran gut microbiota. *Cell Chem. Biol.* **2017**, *24*, 66–75. doi:10.1016/j.chembiol.2016.11.015
33. Chang, P.T.; Rao, K.; Longo, L.O.; Lawton, E.S.; Scherer, G.; Van Arnam, E.B. Thiopeptide defense by an ant's bacterial symbiont. *J. Nat. Prod.* **2020**, *83*, 725–729. doi:10.1021/acs.jnatprod.9b00897
34. Young, T.S.; Walsh, C.T. Identification of the thiazolyl peptide *GE37468* gene cluster from

Streptomyces ATCC 55365 and heterologous expression in *Streptomyces lividans*. *Proc. Natl. Acad. Sci. U. S. A.* **2011**, *108*, 13053–13058. doi:10.1073/pnas.1110435108
35. Bae, M.; Mevers, E.; Pishchany, G.; Whaley, S.G.; Rock, C.O.; Andes, D.R.; Currie, C.R.; Pupo, M.T.; Clardy, J. Chemical exchanges between multilateral symbionts. *Org. Lett.* **2021**, *23*, 1648–1652. doi:10.1021/acs.orglett.1c00068
